# Depression symptoms and the perception of public health restrictions during the COVID-19 pandemic in Saudi Arabia: The protective effect of sense of control

**DOI:** 10.1016/j.pmedr.2022.101836

**Published:** 2022-05-18

**Authors:** Salha Senan, Yemaya Halbrook, Diane E. Kornbrot, Rachel M. Msetfi

**Affiliations:** a King Abdulaziz University, Jeddah, Saudi Arabia; b University of Limerick, University of Limerick, Limerick, Ireland; c University of Hertfordshire, Hatfield, United Kingdom

**Keywords:** Depression, Public health restrictions, Sense of control, COVID-19

## Abstract

The psychological impact of public health restrictions may play a role in the increased depression levels reported since the COVID-19 pandemic began. Data further suggest that people’s sense of personal control has been reduced during the pandemic also producing psychological distress. This study aimed to test whether perceptions of public health restrictions predict depression under pandemic conditions and if the sense of control can serve as a protective factor. For this cross-sectional study, 641 residents in Saudi Arabia over the age of 17 years were recruited between November and December of 2020 to complete an online survey. The survey assessed depression levels (Beck Depression Inventory, BDI), the sense of control and perceptions of public health restrictions. Demographic information was also collected. Depression levels were higher compared to previous samples (*d* = 0.12). The number of restrictions perceived as distressing strongly predicted the probability of high BDI scores, β=0.92, with higher sense of control predicting lower levels of depression (constraints, β=-0.50, mastery, β=-0.71). A strong sense of control significantly reduced the impact of the perception of restrictions on depression. These results suggest that the perception of public health restrictions is part of the reason for increased levels of depression during the pandemic. A strong sense control reduced the impact of restrictions on depression. It is therefore possible that simple interventions enhancing the sense of control, such as the availability of choice, could support mental health in restricted situations.

## 1 Introduction

Since the beginning of the COVID-19 pandemic, concerns have been raised that the public heath restrictions, required to reduce transmission and keep people physically safe, could be a catalyst for mental ill health in general, and especially for depression. The obvious risk factors for depression arising from public health restrictions include protracted social isolation, economic concerns, fears for the safety of elders and so forth. In addition, the removal of every-day freedoms and the unpredictability of the pandemic would also be likely to cause distress (but see, Wang et al., 2020) and induce a reduction in the sense of personal control, another predictor of depression. In this paper, we report a test the hypothesis that people’s perception of public health restrictions, as a source of distress, predicts depression levels. We also test the hypothesis that people’s sense of control will act as a protective factor, reducing the probability of depression.

Due to the seriousness of the pandemic, there is a rapidly accumulating volume of research published, including several reviews. For example, Lakhan et al. (2020) conducted a rapid scoping review of 16 papers published in the first seven months of the pandemic (December 2019 to June 2020). They found that the prevalence of depression, anxiety and stress increased to high levels, in all but one study. A *meta*-analysis of studies published between January and May 2020, reported depression levels to be seven times higher than typically observed (25%, 95% CL: 18% − 33%; Bueno-Notivol et al., 2021).

There is a well-documented relation between sense of control and depression, with low sense of control predicting higher levels of depression (e.g. Bobak et al., 2000, Crandall et al., 2018, Ibrahim et al., 2013, Lachman and Weaver, 1998, Yeo et al., 2017), although we acknowledge that there are a number of other established moderating factors (Moccia et al., 2020). The unpredictability of the pandemic situation would be likely to affect people’s experience of personal control. For example, in many countries public health restrictions were introduced, lifted and re-introduced at short notice, as the virus spread in a number of waves. Along these lines, several studies have now shown that personal control has moderated psychological distress during lockdown (e.g. Brailovskaia and Margraf, 2020, Gan et al., 2020, Xiong et al., 2021). For example, Gan et al. (2020) examined the impact of lockdown on psychological distress in 1390 participants resident in China and found that this was greater in those with lower personal control. As such, we were interested in finding out whether this known moderating factor of a sense of control might protect against the likelihood of depression during covid.

Our previous work has examined relationships between sense of control and depression (Senan et al., 2019) within a population considered to represent a collectivist culture (e.g.. Buda & Elsayed-Elkhouly, 1998), such that the good of the community is paramount (see Triandis, 2001). One might therefore assume that restrictions would have *less* psychological impact within a community that prioritizes the good of the community versus the needs of the individual. However, cultural values are not a unitary characteristic of a nationality or population in one jurisdiction (Green et al., 2005). Some people within a community will express more or less endorsement of collectivistic values whilst others will express more or less endorsement of individualistic values. These values can also be situational such that home and family values are distinct from one's beliefs expressed in a workplace (e.g. Zolfaghari et al., 2016). In addition, recent evidence shows that for countries with similar levels of anxiety, depression and stress prior to the pandemic, significantly different levels were reported in the initial acute stages when public health restrictions such as mask wearing were relatively new (e.g., Wang et al., 2020). For these reasons it is important to measure people’s perceptions of public health restrictions in the same country and examine whether these perceptions influence the established relationship between personal control and measures of depression.

### 1.1 Objectives

In this study, we set out to conduct an exploratory study to examine if people’s perceptions of public health restrictions and the impact on their day-to-day lives would predict their depression levels. We also aimed to examine the impact of personal control as a predictor of depression and the manner in which these two variables interact. The study was located in Saudi Arabia and translated measures of key variables were used as in previous studies (Senan et al., 2019). In addition, for the purposes of this study, we created measures of perception of public health restrictions, in terms of their impact of every-day lives and the extent to which they caused distress. These measures are described below in the method section along with information about the restrictions in place in the country at the time.

## 2 Method

### 2.1 Participants

Following ethical approval, a cross-sectional survey study was conducted in which 641 participants were recruited by online opportunity sampling through University networks, social media, and mobile communication tools, such as WhatsApp. Recruitment continued until no further responses were received on the survey. All participation information was anonymous and recruitment source was not recorded. To be eligible, participants had to declare themselves to be over the age of 17 years and give their consent to participation.

There were 346 female and 295 male participants. The majority (75%) were aged between 18 and 34 years (*n* = 579), 81 reported being 35–44 years, 42 reported being 45–54, 5 reported being 55–64, and 33 reported being 65 or over. Most participants self-reported Saudi nationality (90%, *n* = 577), with the remaining participants self-reporting another Arabic nationality (*n* = 60) and non-Arabic nationality (*n* = 4) respectively.

### 2.2 Measures

Demographic information was collected, including age (years), gender, marital status (single, married, divorced / separated, and widowed), and nationality (Saudi, Resident: Arabic nationals, and Resident: non-Arabic nationals). The survey also asked about participants source of information about COVID-19 (i.e. news release, health media from Saudi Ministry of Health, family and friends, social media, or other), and whether they or their friends or family had been infected by COVID-19 or not.

#### 2.2.1 Beck Depression Inventory (BDI)

The BDI is the primary outcome measure used in this study. It is a well validated, self-report measure of depression, used both in psychiatric and student populations, and has been translated and validated in Arabic (Abdel-Khalek, 1998). The scale consists of 21 items related to depression symptoms and these are scored on a Likert scale from 0 (normal) to 3 (extreme mood), giving a total score from 0 to 63, where higher scores on the questionnaire indicate high levels of depressed mood (Beck et al., 1996). Cronbach’s alpha gave 0.91, showing the scale has high reliability. Standard cut-offs were used to produce a categorical variable where scores of 10 or below represented normal mood (low BDI), and scores of 11 or above indicating some evidence of depression (high BDI).

#### 2.2.2 Sense of control

The Sense of Control scale is a well validated self-report measure of personal control and has been translated and validated in Arabic (see Senan et al., 2019). The scale consists of 12 Likert items rated from 1 (strongly disagree) to 7 (strongly agree). Eight of the items relate to external factors which influence personal control and four items relate to internal factors that influence personal control (Lachman & Weaver, 1998). This gave rise to two distinct but related sub-scale measures of sense of control – perceived constraints and perceived mastery – both of which were obtained by taking the mean of the individual items. Cronbach’s alpha was 0.79 for both subscales.

#### 2.2.3 Public Health Restrictions

When this study was planned, there were no existing validated, published measures that served the purposes of this study. Therefore, we created two measures:

• Restriction impact (0–50): This was the sum of participants’ ratings of the extent to which public health measure affected their (1) daily life, (2) social life, (3) work, (4) daily choices, and (5) weekly choices. Each item was rated from 0 (not at all) to 10 (completely).
• Restriction number: This was the count of how many out of 11 public health restrictions, participants endorsed as making them feel anxious, depressed, or sad. The restrictions were curfew (full curfew / partial curfew), difficulties visiting family / friends, following the required preventive precautions (e.g. wearing masks, sterilizing hands, and measuring temperature before entering any place), fear of getting infected with COVID-19, fear of death, travelling ban, social distance procedures, deterioration in financial state, losing job, losing study, and finally distance learning.

## 3 Procedure

### 3.1 Context

The study was conducted online in the Kingdom of Saudi Arabia, where the government acted swiftly to reduce the spread of the virus (Sayed, 2021). A comprehensive range of restrictions was implemented, with international travel suspended to and from specific locations from the end of January 2020 and general international travel by February 2020 and with students based overseas repatriated. In addition, religious activities were suspended, non-essential venues closed, educational institutions moved online, with a night-time curfew imposed in March 2020. Other measures included social distancing, face covering and sanitizing. As in most jurisdictions, once case numbers dropped, a phased reopening was implemented, with restrictions being reimposed as required to prevent infection spikes. It is clear, however, that public health restrictions have affected most aspects of day-to-day life in Saudi Arabia.

### 3.2 Survey delivery

An online survey was created and hosted on an online platform (Google Forum). The survey remained open from 16th November until 26th December 2020. Volunteers were invited to click on a link in an online advert to participate. They indicated their consent before completing the survey. They were informed that participation was voluntary and they could withdraw at any time by closing the page. All items on the survey were mandatory and participants could not move forward without completing each item.

### 3.3 Analytic Strategy

As all questions for this study were mandatory, there were no missing values. The data were analysed using SPSS Version 28 and the hypotheses were tested using a binary logistic regression model. Descriptive parameters (mean, SD) were obtained for all measures. The binary outcome variable was BDI (low, high) as described above.

The following predictor variables and 2-way interactions were entered into the logistic regression with Forward Selection (Wald): mastery, constraints; restrictions impact, restrictions number, mastery × restrictions impact, mastery × restrictions number, constraints × restrictions impact, constraints × restrictions number. This comprises eight hypotheses.

A post hoc power analysis for a sample of *N* = 641, and alpha = 0.000625 with *df* = 1, gave power 0.99 to detect medium effects and 0.42 to detect small effects. Alpha level was 0.05/8.

Demographic measures are described in the results below but were not entered into the analysis. Note that the bivariate correlations between the predictor variables were checked for multicollinearity, all *r* < 0.28, prior to conducting the regression analyses.

## 4 Results

### 4.1 Demographic measures

Many participants reported that their main source of information about Covid-19 was derived from the Ministry of Health announcements (58%), whilst, for others, information mainly came from social media (17%), news releases (15%) and from family and friends or other sources. Only 66 participants reported having been infected with COVID-19 (10%), though 432 said that a family member or friend had been infected (67%). In terms of restrictions that participants found distressing, the most common finding was that curfew (50.7%) and difficulties visiting family (51.6%) were distressing.

### 4.2 Descriptive measures

The average BDI score in this sample was 13.20 (*SD* = 11.11) and was significantly higher than our previous work using the same translated version of the BDI where scores were *M* = 11.88, *SD* = 9.48, single samples *t*(6 4 0) = 3.00, *p* = 0.001, Cohen’s *d* = 0.12. 46% of participants in the current study scored in the high BDI category (*n* = 295). Mean scores on the sense of control subscales were calculated, with constraints producing an average score of 4.10, *SD* = 1.21, and mastery producing an average of 4.92, *SD* = 1.51. Table 1 further shows shows descriptive statistics for all the predictor variables as a function of low and high BDI group, with participants in the high BDI category tending to produce lower scores on the sense of control sub-scales, and higher scores on the measures of perception of public health restrictions.

**Table 1:** Summary of predictor variable means for low and high BDI participants.

BDI category	Predictor Variables	*M*	*SD*	*95%* *LCL*	*95%* *UCL*
Low BDI	Mastery	5.35	1.32	5.20	5.50
	Constraints	4.64	1.05	4.53	4.75
	Restriction Impact	30.06	12.95	28.81	31.31
	Restriction Number	3.05	1.86	2.85	3.25
High BDI	Mastery	4.41	1.56	4.24	4.57
	Constraints	3.47	1.09	3.35	3.59
	Restriction Impact	34.12	10.49	32.76	35.48
	Restriction Number	3.47	1.95	3.25	3.69

Data are mean values (M) and standard deviations (SD) with lower and upper 95% confidence limits (LCL, UCL).

### 4.3 Inferential statistics

We tested the hypotheses using binary logistic regression. Table 2 shows the results for all variables with a significant predictive effect on BDI group.

**Table 2:** Binary logistic regression model for BDI (low, high) with Forward Selection.

**Variables**	***B***	***SE***	***Wald***	***df***	***p***	**Exp(*B*)**	**ESw**
**1. Restrictions number**	0.92	0.27	12.09	1	0.0005	2.52	0.15
**2. Constraints**	−0.50	0.19	6.86	1	0.0088	0.60	0.10
**3. Mastery**	−0.71	0.10	48.70	1	<0.00005	0.49	0.30
**Mastery I Restrictions Impact**	0.01	0.01	9.09	1	0.0026	1.01	0.12
**Constraints I Restrictions number**	−0.19	0.06	9.43	1	0.0021	0.82	0.13
**Constant**	4.17	0.98	18.26	1	<0.001	64.37	

NB. The interaction between variables is indicated by the I symbol. The effect size measure w is referred to as ESw and was calculated as √ (Wald/N).

**Fig. 1:**
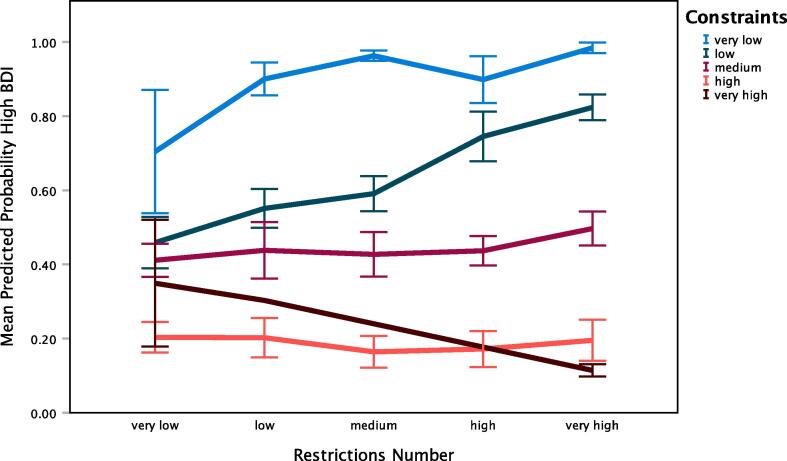

**Fig. 2:**
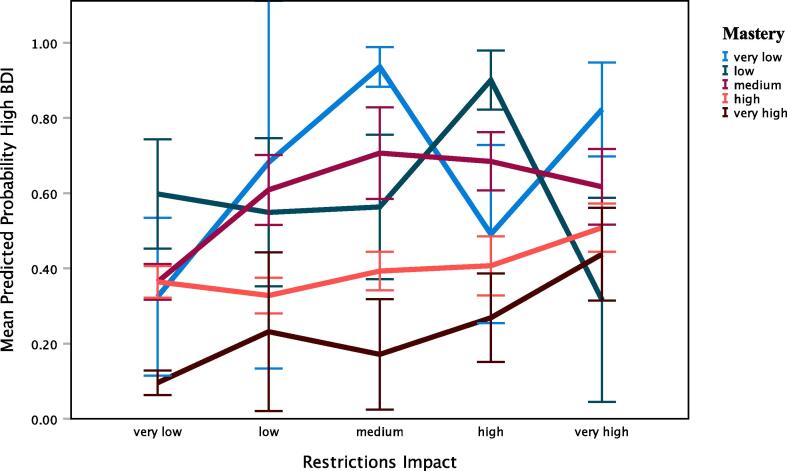

This analysis shows that a higher number of restrictions, cited as distressful, predicted a higher probability of high BDI status. In addition, with both sense of control measures, mastery and constraints, a higher sense of control was a significant predictor of low BDI status. There were two significant two-way interactions, between mastery and restrictions impact, and constraints and restrictions number. As the analysis was conducted on continuous predictor variables, these were split into categories with five levels to aid interpretation (see Fig. 1, Fig. 2).

Fig. 1 shows that the extent to which a high number of restrictions predicted high BDI category depended on constraints. The interaction shows that with a high or very high sense of control (constraints), higher restriction numbers did not significantly increase the likelihood of high BDI scores, mean probability (high BDI) = 0.24 for all restriction numbers. By contrast, for participants with the lowest sense of control, the likelihood of high BDI increased by 32.7% with restriction numbers.

Fig. 2 shows that increasing restrictions impact was also predictive of high BDI category. The interaction shows that this effect was more evident in those with low or very low sense of mastery. The highest *p* (BDI high) values were observed in those with the lowest sense of control (mastery) with the highest impact of restrictions scores (*p* = 0.78, very low mastery; *p* = 0.84, low mastery). For those with the highest sense of control, *p* high BDI increased to *p* = 0.50 (high mastery) and *p* = 0.43 (very high mastery) with the highest level of impact.

## 5 Discussion

When participants perceived higher numbers of public health restrictions to be distressful, they were more likely to show evidence of depression. This is a new finding. Perception of restrictions has not previous been studied quantitatively in relation to depression. Having a stronger sense of control was predictive of lower levels of depression as has been found elsewhere. Importantly, sense of control was a moderator of the effect of restrictions, generating two new findings. Those with a high sense of control, expressed through minimal perceived constraints were less likely to be depressed with high numbers of distressful restrictions. Those with a high sense of control expressed through strong perceived mastery were less likely to be depressed with high impact restrictions. These findings are consistent with the idea that having a strong sense of personal control can protect the individual from the deleterious impact of public health restrictions.

The findings of this study, conducted in Saudi Arabia, are consistent with other studies across the world conducted since the pandemic began. Similar to previous studies, (e.g. Bueno-Notivol et al., 2021, Choi et al., 2020, Hyland et al., 2020), we found evidence of elevated levels of depression in comparison to pre-pandemic levels. For example, Ettman et al. (2020) found a three-fold increase in depression symptoms in the US when comparing depression levels before and during the pandemic.

Whilst the real threat to life and health would almost certainly create fear and distress, it has also been argued that protective public health restrictions themselves would also have a negative psychological impact (Holmes et al., 2020). We explored that hypothesis in this study. We found that the number of restrictions that participants indicated caused them distress that were predictive of depression. The important result here however is that sense of control moderated the deleterious effect of restrictions.

Specifically, the sense of personal control appeared to act as a strong protective factor during the pandemic. Those with a strong sense of control, through a strong feeling of mastery or a feeling that they, rather external factors (perceived constraints), control their lives, were less likely to score high on the depression scale. Importantly, perceived constraints (e.g., “What happens in my life is often beyond my control” etc.,) moderated the effect of number of restrictions, such that those who perceived less in terms of external constraints were not likely to be influenced by number of restrictions. Mastery had a similar moderating effect (e.g., “I can do just about anything I really set my mind to”) on the impact of restrictions. As we have reported elsewhere, the perceived external sources of control (context, external forces) are an important aspect of measuring the sense of control (Lachman & Weaver, 1998) and a modifier of perceived control and been used to enhance the sense of control in the laboratory (Msetfi et al., 2016).

These findings add to considerable evidence that is consistent with the protective role of the sense of personal control in depression, (e.g. Bobak et al., 2000, Crandall et al., 2018, Ibrahim et al., 2013, Lachman and Weaver, 1998) irrespective of the cultural values of the population tested. When planning this study, we thought that for people embedded in cultures in which the common good is prioritised over individual benefit, collectivitism versus individualism; (see Triandis, 2001) and might be habituated to a degree of social restriction in comparison to many western cultures (e.g., face covering is normative for women in Saudi Arabia), public health restrictions might not cause such psychological distress as in other jurisdictions. This was not the case. People in this sample showed higher levels of depression than in pre-COVID-19 studies (Senan et al., 2019), with the number of restrictions, perceived being as distressful, being a predictive factor for depression. The key new finding in this study is that a strong sense of control reduced the effect of restrictions on depression likelihood, which has the potential to contribute to our understanding of the effectiveness of psychological interventions designed to support people during such challenging times (Ho et al., 2020).

### 5.1 Limitations

This study is not without limitations. Firstly, all measures were self-report, and collected though an online forum. Whilst there were distinct differences between this data set and previous data collected from a similar population using the same measures, this methodology does not provide longitudinal pre and post COVID-19 levels of depression. In addition, whilst these data are consistent with the significant body of evidence supporting the protective nature of the sense of control in relation to depression and psychological distress (Lachman & Weaver, 1998), this study is correlational and causal conclusions are not possible. Finally, the study was conducted during a time of uncertainty about COVID-19 and restrictions being reimposed as cases began to increase prior to the January 2021 spike in cases. Therefore, these findings do not speak to the immediate crisis of March 2020 and its immediate psychological impact. Rather, this study and it's timing speaks to the medium-term consequences.

Findings are correlative rather than causative. It may be that people with low level for depression are less likely to see restrictions as distressful and more likely to have a sense of control. This is an inevitable limitation of such convenience sampling cross-sectional studies.

## 6 Conclusion

The people who participated in this study experienced the COVID-19 pandemic in the context of public health measures in the Kingdom of Saudi Arabia in the Autumn of 2020. As in many other countries, findings indicated increased levels of depression than before the pandemic. People’s experiences of public health restrictions, both impact and number, predicted depression levels. This is a new finding. Importantly, a strong sense of control was a protective factor against the likelihood of depression and mitigated the harmful psychological effect of public health restrictions. Evidence shows that simple interventions can enhance the sense of control (Thompson, 2020). Consequently, procedures that increase sense of control, such as providing choice where appropriate (Xu et al., 2020), have substantial potential to mitigate the effects of adverse events, not just COVID-19. Further studies might usefully explore how the nature and content of messaging in the public health domain could alter perceptions and usefully leverage these findings.

## Funding

This project was funded by the Deanship of Scientific Research (DSR) at King Abdulaziz University, Jeddah, under grant No. (D-070-246-1441), Principal Investigator Dr. Salha Senan, Co-Investigator Prof. Rachel Msetfi.

## 8 Ethical Standards

The authors assert that all procedures contributing to this work comply with the ethical standards of the relevant national and institutional committees on human experimentation and with the Helsinki Declaration of 1975, as revised in 2008.

## Authors contributions

SS engaged in data curation, investigation, methodology, and writing (review and editing). YH engaged in formal analysis, project administration, methodology, data curation, and writing (review and editing). DEK engaged in formal analysis, visualisation, methodology, and writing. RMM conceptualized the study, conducted literature searches, study design, selection of measures, data analysis, data interpretation, and writing. SS and RMM engaged in funding acquisition. All authors directly accesses and verified the underlying data reported.

## 10 Data sharing

Data collected for this study will be available to other researchers for download following publication. Msetfi, Rachel (2021), “Public health restrictions and depression”, Mendeley Data, v1http://dx.https://doi.org/10.17632/bk7j8rs5rj.1DOI is reserved but not active.

## Declaration of Competing Interest

The authors declare that they have no known competing financial interests or personal relationships that could have appeared to influence the work reported in this paper.
